# Vertebrobasilar Artery Calcification as a Risk Factor for the Development of Stroke: Case-Control Study From a Tertiary Care Center, Southern India

**DOI:** 10.7759/cureus.42196

**Published:** 2023-07-20

**Authors:** Kibballi M Adarsh, H Pavithra, Valiyapalathingal Abdul Rasheed

**Affiliations:** 1 Radiodiagnosis and Medical Imaging, Yenepoya Medical College Hospital, Mangaluru, IND; 2 Community Medicine, Yenepoya Medical College, Mangaluru, IND

**Keywords:** vertebrobasilar artery calcification, computed tomography, brain, stroke, non-communicable diseases

## Abstract

Background

Stroke is an important cause of morbidity and mortality in developing countries. Many risk factors are well-established for the development of stroke. Atherosclerosis involving the media and intima layer of vertebrobasilar arteries has not been studied well as a risk factor for stroke.

Objective

To assess the degree of calcification of vertebrobasilar arteries among patients with stroke (cases), age- and gender-matched controls, and the risk of the development of stroke.

Methods

This was a hospital record-based case-control study with individuals with stroke as cases and age- and gender-matched individuals without stroke as controls, who underwent computed tomography imaging. The degree of calcification of vertebrobasilar arteries was assessed among cases and controls. Various other risk factors for the development of stroke such as the presence of hypertension, diabetes mellitus, and impaired lipid profile were also assessed.

Results

Among the 150 cases and their age- and gender-matched controls, the mean age of the subjects was 58.38 (±14.06) years. Forty percent of the individuals were males. Among the cases, right vertebral, left vertebral, and basilar artery calcifications were observed among 114 (76%), 102 (68%), and 52 (34.7%), respectively. Among controls, 71 (47.3%), 77 (51.3%), and 29 (19.3%) individuals had right vertebral, left vertebral, and basilar artery calcifications, respectively. The degree of calcification was higher among the cases in the right and left vertebral arteries than the controls (adjusted odds (aOR) of 6.61 and 2.32 and p-value <0.001 and 0.032, respectively). The risk of stroke increased with higher degrees of calcification, the presence of diabetes mellitus, and raised cholesterol levels (aOR of 9.98, 2.32, and 4 and p-value <0.001, 0.007, and 0.001, respectively). The risk of stroke increases with the presence of multiple risk factors.

Conclusion

The presence of higher grades of calcifications in the vertebrobasilar arteries is a major risk factor for stroke. Hence, its presence in asymptomatic individuals needs to be taken as a warning sign for the development of stroke.

## Introduction

The World Health Organization (WHO) defines stroke as rapidly developing clinical signs of focal disturbance of cerebral function, lasting more than 24 hours or leading to death, with no apparent cause other than vascular origin. This disturbance of brain function can be caused by stenosis, rupture, or occlusion of the arteries [1]. The definition of transient ischaemic attack (TIA) is that they last for less than 24 hours [2]. Being a worldwide health problem, stroke is an important contributor to morbidity, disability, and mortality in developing as well as developed nations. According to the Global Burden of Diseases (GBD), stroke contributed to 5.5 million deaths and 116.4 million disability-adjusted life years (DALYs) in the year 2016. In the same year, 13.7 million new cases were identified. Though there is a decrease in the age-standardized mortality rate of stroke, better survival makes it one of the leading causes of morbidity. Around 1.1 million incident cases and 16.3 million DALYs due to stroke were reported in 2016 in India [3]. It is estimated that 86.5 deaths/ lakh population in India are caused by stroke [4]. 

Many risk factors for stroke are well-established. Diseases such as hypertension, diabetes mellitus, sedentary lifestyle, smoking, and alcohol consumption are very well implicated in the causation of stroke, along with non-modifiable risk factors such as age and gender [5]. However, there are a few other risk factors, including intracranial atherosclerosis [6]. 

The atherosclerosis of intracranial arteries can be observed in the intimal and medial layers. Medial calcifications can be caused by varied reasons such as genetic disorders, chronic kidney disease, diabetes mellitus, dyslipidemia, etc. Intima calcifications are usually due to inflammatory cytokines, metabolic syndrome, and hyperlipidemia [7]. The presence of diabetes is known to increase the risk of atherosclerosis. This is mainly due to endothelial dysfunction and inflammation due to the state of hyperglycemia [8]. Atherosclerosis of intracranial arteries has not been well-studied as the coronary arteries. The reasons for the same can be the infrequency of intracranial atherosclerosis in races such as Caucasians and the poor accessibility of these arteries. The degree of calcification has been found to vary with ethnicity [9]. The atherosclerotic plaque has various components such as the cells, the connective tissue of the extracellular matrix, and the intra and extra-cellular lipid deposits. The presence of calcification in an atherosclerotic plaque indicates an advanced or complex form of atherosclerosis. These calcifications are observed in 40-60% of ischaemic stroke patients undergoing computed tomography (CT) scans [10]. 

This study was thus conducted to assess the degree of vertebrobasilar calcification among patients with stroke (cases) and age- and gender-matched controls and the risk of the development of stroke.

## Materials and methods

This case-control study was conducted in a medical college teaching tertiary care hospital located in the coastal city of Karnataka, India. It caters to about 31 districts and approximately 68.4 million population [11]. We included data available from January 2018 till December 2019. All the cases referred to the department of radio-diagnosis for CT imaging during this study period were considered as the study population from which the cases and controls were selected. The cases and controls were age (± 3 years) and gender-matched in a 1:1 ratio.

Waiver of consent was obtained from the institutional ethics committee (protocol number 2020/048, dated 31/08/2020) as this was a record-based retrospective study. The radiological data of cases and controls were obtained from the picture archive and communication system (PACS) of the institution. The data regarding the co-morbidities were collected from hospital files and obtained from the medical records department (MRD). Our cases were individuals with radiological features of stroke, and controls were individuals without stroke, specifically the patients who underwent CT scans for indications other than stroke. These included patients with chronic headaches, psychiatric illnesses, etc. The details of the cases and controls were obtained from the hospital data.

We used Openepi software to calculate the sample size. Considering an odds ratio of two [12], we estimated the sample size to be 134 subjects each for cases and controls, and to this, we added 10% of the patients with stroke that might not undergo a CT scan. Thus, our final sample size was 150 each for cases and controls. A pre-designed, validated questionnaire was used for data collection. The data were captured using Epicollect5 software. Epicollect5 is open-access software with a web portal and smartphone/tablet application. The web portal is for questionnaire building, downloading the entered data, and other features, whereas the application is for data capture. CT brain images of all the individuals that were imaged during the study period were reviewed. A 16-slice GE Pioneer CT machine was used for imaging, and the images were reconstructed to 2.5 mm thickness on the brain window and bone window.

The CT scans of the study period were accessed and screened for the presence of stroke (cases) till the desired sample size was reached. Only the individuals having an acute stroke were included in this study. Both territorial and lacunar infarcts were included. Individuals having hemorrhagic and old strokes were excluded. The CT images were retrieved from a picture archiving and communication system (PACS). Their age and gender-matched controls were further selected from PACS, belonging to the same study period. Bilateral vertebral and basilar arteries were evaluated for the degree of wall calcification. The part of the vertebral artery that was considered for assessing the calcification was from the dura at the lateral edge of the posterior atlanto-occipital membrane to their confluence on the medulla to form the basilar artery. The basilar artery was considered from its origin (confluence of the vertebral arteries) till its division into the two posterior cerebral arteries [6]. 

**Figure 1:**
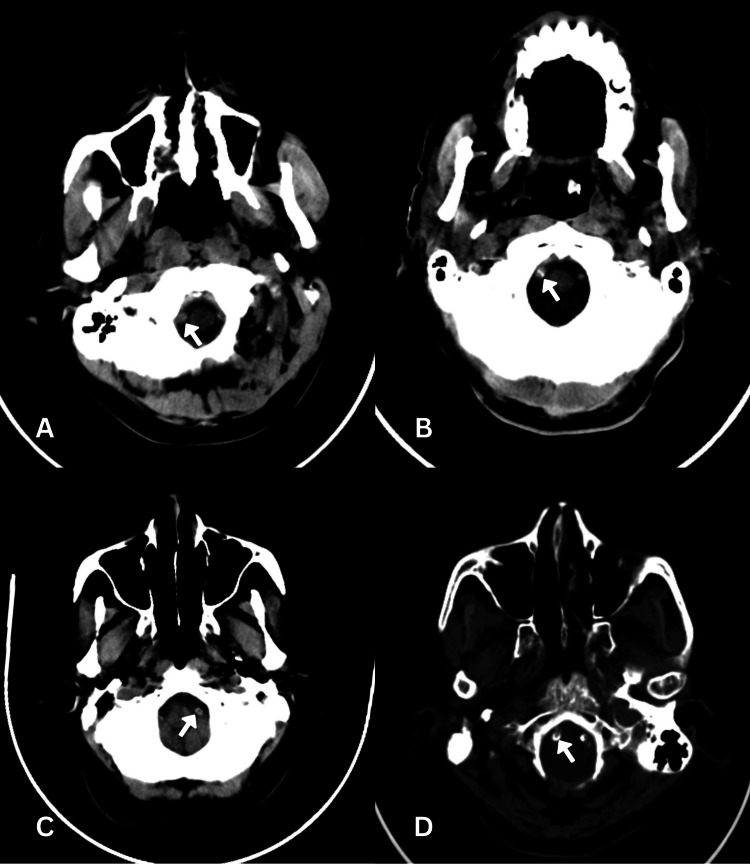
Examples of different grades of vertebrobasilar artery calcification (A) Grade 1: Dot-like calcification in any one quadrant. (B) Grade 2: Focal type of calcification, not more than 1/4th of the circumference or multiple small foci of calcification in the same segment, accounting for not more than 1/4th of the circumference. (C) Grade 3: Crescent calcification extending over 1/4th, but not through the entire circumference. (D) Grade 4: Circular - entire vessel circumference calcification

Vertebral arteries were divided into four quadrants: ventral medial, ventral lateral, dorsal medial, and dorsal lateral. The density of >90 Hounsfield units (HU) was considered calcification [13] (Figure 1).

The wall calcifications were graded on an axial set of images as follows:

Grade 0: No calcification throughout the artery.

Grade 1: Dot-like calcification in any one quadrant.

Grade 2: Focal type of calcification, not more than 1/4th of the circumference or multiple small foci of calcification in the same segment, accounting for not more than 1/4th of the circumference.

Grade 3: Crescent calcification extending over 1/4th, but not through the entire circumference (25-100%).

Grade 4: Circular - entire vessel circumference [6]. 

In an artery, when there were multiple sections with wall calcifications, the section with the maximum circumference of calcifications was considered. Calcifications seen in more than one section on axial images were termed vertical dispersion. Vertical dispersions of the calcifications were evaluated irrespective of the type of calcification [6]. The degree of maximum stenosis was calculated by measuring the area of calcification v/s total vessel area and was expressed as a percentage. The basilar artery was divided into four quadrants: ventral right, ventral left, dorsal right, and dorsal left for evaluation. The rest of the calcification grading was done similarly to that of the vertebral artery.

The details of the other risk factors for stroke such as diabetes mellitus, hypertension, and abnormal lipid profile of cases and controls were obtained from patient files and archived in the medical records department of the institution. Case files with incomplete data on the risk factors were excluded from the analysis.

The data were analyzed using Statistical Package for Social Sciences (SPSS) software (IBM Corp. Released 2015. IBM SPSS Statistics for Windows, Version 23.0. Armonk, NY: IBM Corp.). Descriptive statistics were expressed as mean and standard deviation. Binary logistic regression was performed to assess the odds of developing stroke with the presence of vertebrobasilar artery (VBA) calcification and various other risk factors.

## Results

This study had 150 cases and 150 age- and gender-matched controls. The mean age of the subjects was 58.38 (±14.06) years, with the minimum and maximum ages recorded being 16 and 85 years, respectively. Out of the 300 individuals, 120 (40%) were males. Among the cases, three (1%), 100 (33.3%), and 28 (9.3%) had anterior, middle, and posterior cerebral artery strokes, respectively. Moreover, three (1%) had both anterior and middle cerebral artery strokes, and 16 (5.3%) had both middle and posterior cerebral artery strokes. A total of 12 infratentorial infarcts were observed among cases. Out of these, eight infarcts were present, along with posterior cerebral artery stroke, and the rest were present with middle cerebral artery stroke.

The grading of calcification was done in the vertebral artery (right and/or left) and basilar artery. The calcifications were divided into single focus and multiple foci, when present. The single-focus calcifications were further assessed for the type of calcification and were graded 1-4. (Tables 1-3). Multiple-focus calcifications were further divided into pure dot/pure grade 2/pure grade 3/pure grade 4/grades 1 and 2/grades 3 and 4/grades 2 and 4/grades 1, 2, and 3/grades 2, 3, and 4/all types (Tables 1-3).

**Table 1:** Details of calcifications in the right vertebral artery in cases and controls, among patients of a tertiary care hospital, Southern India (2018-19, N=300)

Type of calcification in the right vertebral artery	Cases, n (%)	Controls, n (%)
No calcification	36 (24)	79 (52.7)
Calcification present	114 (76)	71 (47.3)
Single-focus calcification	8 (5.3)	40 (26.7)
Multifocal calcification	106 (70.7)	31 (20.6)
Type of single-focus calcification, n = 8		
Grade 1/dot of calcification	4 (2.7)	35 (23.3)
Grade 2/focal calcification 90 degrees spanning the wall of the artery	2 (1.3)	5 (3.3)
Grade 3/crescent 1/4^th^ to 3/4^th^ of the wall of the artery	2 (1.3)	0 (0)
Grade 4/circular	0 (0)	0 (0)
Type of multifocal calcification, n = 106		
Pure type	25 (16.7)	6 (4)
Mixed type (2 types)	48 (32)	20 (13.4)
Mixed type (3 types)	30 (20)	5 (3.3)
All types	3 (2)	0 (0)

**Table 2:** Details of calcifications in the left vertebral artery in cases and controls, among patients of a tertiary care hospital, Southern India (2018-19, N=300)

Type of calcification in the left vertebral artery	Cases, n (%)	Controls, n (%)
No calcification	48 (32)	73 (48.7)
Calcification present	102 (68)	77 (51.3)
Single-focus calcification	8 (5.3)	43 (28.7)
Multifocal calcification	94 (62.7)	36 (24)
Type of single-focus calcification, n = 8		
Grade 1/dot of calcification	5 (3.3)	36 (24)
Grade 2/focal calcification 90 degrees spanning the wall of the artery	2 (1.3)	7 (4.7)
Grade 3/crescent 1/4^th^ to 3/4^th^ of the wall of the artery	1 (0.7)	0 (0)
Grade 4/circular	0 (0)	0 (0)
Type of multifocal calcification, n = 94		
Pure type	10 (6.7)	5 (3.3)
Mixed type (2 types)	52 (34.7)	17 (11.3)
Mixed type (3 types)	22 (14.7)	14 (9.3)
All types	6 (4)	0 (0)

**Table 3:** Details of calcifications in the basilar artery in cases and controls, among patients of a tertiary care hospital, Southern India (2018-19, N=300)

Type of calcification in the basilar artery	Cases, n (%)	Controls, n (%)
No calcification	98 (65.3)	121 (80.7)
Calcification present	52 (34.7)	29 (19.3)
Single-focus calcification	6 (4)	23 (15.3)
Multifocal calcification	46 (30.7)	6 (4)
Type of single-focus calcification, n = 6		
Grade 1/dot of calcification	2 (1.3)	19 (12.7)
Grade 2/focal calcification 90 degrees spanning the wall of the artery	4 (2.7)	4 (2.7)
Grade 3/crescent 1/4^th^ to 3/4^th^ of the wall of the artery	0 (0)	0 (0)
Grade 4/circular	0 (0)	0 (0)
Type of multifocal calcification, n = 46		
Pure type	11 (7.3)	0 (0)
Mixed type (2 types)	16 (10.7)	3 (2)
Mixed type (3 types)	19 (12.7)	3 (2)
All types	0 (0)	0 (0)

For binary logistic regression, grades 1 and 2 of the calcifications were grouped as "lower grade," and grades 3 and 4 were grouped as "higher grade." Different characteristics of the calcification such as the grade of calcification, multifocal calcification, and vertical dispersion for each artery were further subjected to regression analysis. For both the vertebral arteries, a higher grade of calcification was found to have a greater risk of stroke development, whereas, for the basilar artery, vertical dispersion had higher odds for the development of stroke (Table 4).

**Table 4:** Association between the characteristics of calcification in vertebral and basilar arteries and stroke among patients of a tertiary care hospital, Southern India (2018-19, N=300) OR: Odds ratio; aOR: adjusted odds ratio; *: statistically significant association

Vertebrobasilar arteries		Cases, n (%)	Controls, n (%)	Crude OR (95% CI)	p-value	aOR	p-value
Right vertebral artery							
Grade of calcification	Higher	69 (46)	7 (4.67)	17.4 (7.63–39.66)	<0.001^*^	6.61 (2.61–16.71)	<0.001^*^
	Lower	81 (54)	143 (95.33)				
Multifocal calcification	Yes	106 (70.67)	31 (20.67)	9.25 (5.44–15.69)	<0.001^*^	3.67 (0.72–18.64)	0.117
	No	44 (29.33)	119 (79.33)				
Vertical dispersion of the calcification	Yes	103 (68.67)	29 (19.33)	9.14 (5.37–15.57)	<0.001^*^	1.2 (0.23–6.40)	0.827
	No	47 (31.33)	121 (80.67)				
Left vertebral artery							
Grade of calcification	Higher	92 (61.33)	15 (10)	5.67 (3.03–10.62)	<0.001^*^	2.32 (1.07–5.02)	0.032^*^
	Lower	58 (38.67)	135 (90)				
Multifocal calcification	Yes	94 (62.67)	36 (24)	5.31 (3.22–8.76)	<0.001^*^	1.34 (0.07–23.84)	0.841
	No	56 (37.33)	114 (76)				
Vertical dispersion of the calcification	Yes	93 (62)	35 (23.33)	5.36 (3.24–8.86)	<0.001^*^	2.63 (0.15–46.15)	0.506
	No	57 (38)	115 (76.67)				
Basilar artery							
Grade of calcification	Higher	29 (19.33)	2 (1.33)	17.74 (4.15–75.83)	<0.001^*^	1.90 (0.23–15.71)	0.550
	Lower	121 (80.67)	148 (98.67)				
Multifocal calcification	Yes	45 (30)	5 (3.33)	12.43 (4.77–32.38)	<0.001^*^	0.53 (0.04–6.59)	0.623
	No	105 (70)	145 (96.67)				
Vertical dispersion of the calcification	Yes	45 (30)	3 (2)	21 (6.36–69.39)	<0.001^*^	27.22 (1.89–391.22)	0.015^*^
	No	105 (70)	147 (98)				

 Various risk factors were analyzed using binary logistic regression. Having higher grades of calcification, the presence of diabetes mellitus and raised cholesterol levels were found to increase the risk of stroke development (Table 5).

**Table 5:** Association between risk factors and the presence of stroke among patients of a tertiary care hospital, Southern India (2018-19, N=300) OR: Odds ratio; aOR: adjusted odds ratio; *: statistically significant association; TIA: transient ischemic attack; HDL: high-density lipoprotein

Risk factors		Cases, n (%)	Controls, n (%)	Crude OR (95% CI)	p-value	aOR (95% CI)	p-value
Vertebrobasilar artery calcification	Yes	127 (84.67)	93 (62)	3.38 (1.95–5.88)	<0.001^*^	1.51 (0.77–3.00)	0.232
	No	23 (15.33)	57 (38)				
Grade of vertebrobasilar artery calcification	Higher	89 (59.33)	19 (12.67)	10.06 (5.63–17.99)	<0.001^*^	9.98 (4.89–20.34)	<0.001^*^
	Lower	61 (40.67)	131 (87.33)				
Hypertension	Present	93 (62)	60 (40)	2.44 (1.53–3.89)	<0.001^*^	1.04 (0.57–1.89)	0.894
	Absent	57 (38)	90 (60)				
Diabetes mellitus	Present	100 (66.67)	89 (59.33)	1.37 (0.86–2.19)	0.189	2.32 (1.25–4.31)	0.007^*^
	Absent	50 (33.33)	61 (40.67)				
Previous history of TIA	Present	17 (11.33)	46 (30.67)	0.29 (0.16–0.53)	<0.001^*^	0.19 (0.09–0.41)	<0.001^*^
	Absent	133 (88.67)	104 (69.33)				
HDL levels	Lowered	92 (61.33)	105 (70)	0.68 (0.42–1.09)	0.115	0.57 (0.31–1.03)	0.062
	Normal	58 (38.33)	45 (30)				
Total cholesterol levels	Raised	33 (22)	12 (8)	3.24 (1.60–6.57)	0.001^*^	4 (1.73–9.2)	0.001^*^
	Normal	117 (78)	138 (92)				

**Figure 2:**
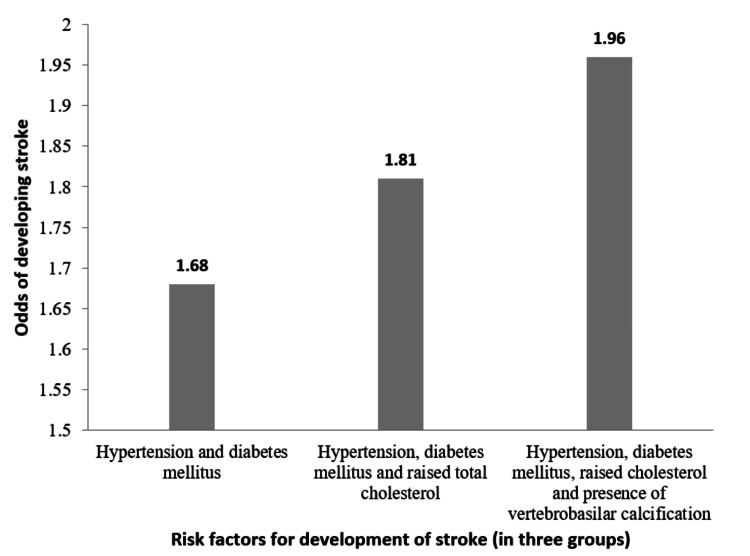
Odds of developing stroke with the presence of various risk factors among patients of a tertiary care hospital, Southern India (2018-19)

The presence of various co-morbidities and their influence on stroke causation was clubbed, and the findings are presented in Figure 2. 

## Discussion

This was a case-control study done to assess the degree of VBA calcification and its association with stroke. The cases and controls were age- and gender-matched. As stroke has multi-factorial causation, other factors such as hypertension, diabetic status, and lipid profile of the participants were considered confounders.

The mean age of the participants was 58.38 (±14.06) years in our study. Another study conducted in India had 58.3 ± 14.7 years as the mean age of stroke cases [14]. Other similar studies had patients of ages 67.7 (±12.8) years among Korean ethnicity [10] and 76 (67.7-87.2) years, 69.2 ± 6.8 years, and 69.8 (± 10.6) years among Caucasians [13,15,16]. When compared to these studies conducted in other parts of the world, our study participants had considerably lower ages for incidence of stroke. With developing countries reporting decreased mean age for the development of stroke, the economic burden to be faced by the individual and families has substantially increased. The economic costs involved include direct costs related to the medications and treatment and indirect costs related to loss of employment [17].

VBA calcification was present among 84.67% of cases and 62% of the controls in our study. In a study that analyzed the ischemic stroke profile, its risk factors, and its outcomes in India, arterial occlusion of >50% stenosis was found in 42.7% of the study participants. Further, VBA had occlusion of >50% stenosis among 7.5% of the cases, indicating higher grades of stenosis. This study thus confirmed higher rates of intracranial atherosclerosis [14]. Another study on a hospital-based stroke registry in India found that 37.6% of the cases belonged to the large artery atherosclerosis subtype with a predominance of intracranial atherosclerosis [18]. In another study conducted among Caucasians, vertebrobasilar calcifications were among 52.56% of the cases with ischemic stroke [6]. Any form of intracranial artery calcification was found in 61.4% of the cases in the study conducted among the Korean population [8]. A study among Caucasians had a 21% overall prevalence of VBA calcification among the general population [15]. Hence, VBA calcification is more prevalent in the Indian population compared to the others. It is notable that the presence of calcifications among control groups was also higher in our study.

The calcifications of the right and left vertebral arteries were further characterized as single-focus or multifocal calcifications. Cases were found to have calcifications more frequently than the controls in all three arteries. Among cases, the majority of the calcifications were of multiple foci in all three arteries, whereas the control group had a majority of single-focus calcifications. The presence of higher grades of calcification, multifocal calcifications, and vertical dispersion of calcification was found to have higher odds of the development of stroke in our study. Another study found that focal calcifications presented more frequently compared to other types of calcifications in their study [6]. 

The association of stroke with various other known risk factors was analyzed. The presence of diabetes and raised total cholesterol levels had higher associations with the development of stroke. The adjusted odds ratio were 2.32 and 4, respectively, and these associations were statistically significant (p <0.001). A case-control study conducted on risk factors for the development of stroke found that the risk of stroke increased with the presence of hypertension and diabetes by 2.64 and 1.36 times, respectively [19]. The presence of a previous history of TIA had odds of 0.19 for the development of stroke, indicating that treatment following one episode of TIA was effective against the recurrence of further episodes. This association was statistically significant (p <0.001). However, the presence of higher grades of vertebrobasilar calcification had the highest risk of the development of stroke with an adjusted odds ratio of 9.98 (p <0.001).

The risk factors were further clubbed to obtain the odds of the development of stroke when they are present together in an individual. The presence of both diabetes and hypertension had odds of 1.69 (p <0.001) and the presence of raised total cholesterol along with diabetes and hypertension had odds of 1.81 (p <0.001) for the development of stroke. When VBA calcification was present along with these three risk factors, the odds were found to be 1.96 (p <0.001) (Figure 2). When presented with other known risk factors of stroke, the presence of VBA calcification increased the risk of stroke. When considered as a single risk factor, VBA calcification had 3.38 higher odds of stroke development (p <0.001).

The strengths of our study are that the literature regarding VBA calcification among stroke patients is limited, and this study adds to the evidence. We employed a case-control design to conduct this study with age- and gender-matched controls. Other disease conditions that are established risk factors for the development of stroke were also adjusted for in the analysis. The study also had a large sample size. However, this being a hospital record-based retrospective study, an important limitation was the poor availability of certain important confounders, such as smoking and alcohol consumption and level of physical activity, as these were not accurately mentioned. Additionally, the severity of co-morbid conditions, such as diabetes and hypertension, was not very well captured in the patient files. Another limitation of this study is that the data on internal carotid artery calcifications were not captured. As these calcifications can also act as a risk factor for stroke, the inclusion of the same would have provided more insight regarding intracranial calcifications and the occurrence of stroke. The other limitation could be that CT brain plain was considered for the study, rather than CT angiography. We would like to mention here that diagnosis of stroke is done primarily using CT brain plain studies only in the present study setting, as all patients cannot afford CT angiography.

## Conclusions

This was a case-control study conducted to find the association between vertebrobasilar artery calcification and the risk of the development of stroke. The presence of higher grades of VBA calcification had 9.98 higher odds of developing stroke. When VBA was present, along with the traditional risk factors for stroke such as diabetes mellitus, hypertension, and impaired lipid profile, it increased the risk of the occurrence of stroke. Thus, VBA calcification is one of the risk factors for the occurrence of stroke, and the presence of VBA in asymptomatic individuals needs to be considered as a warning sign for the development of stroke. The development of a scoring criterion for VBA calcification in the standard reporting format of brain CTs can be a first step toward the quantification of this risk factor. This can help in highlighting the increased risk of the development of stroke and further help the treating physicians to control this risk factor.
